# Proinflammatory Role of Angiotensin II in the Aorta of Normotensive Mice

**DOI:** 10.1155/2019/9326896

**Published:** 2019-01-27

**Authors:** Rariane Silva de Lima, Juliane Cristina de Souza Silva, Cintia Taniguti Lima, Leandro Ezequiel de Souza, Maikon Barbosa da Silva, Marina Gazzano Baladi, Maria Claudia Irigoyen, Silvia Lacchini

**Affiliations:** ^1^Department of Anatomy, Institute of Biomedical Sciences, University of São Paulo, Brazil; ^2^Hypertension Unit, Heart Institute, University of São Paulo Medical School, Brazil

## Abstract

Angiotensin II plays important functions in cardiovascular system mediating actions leading to inflammatory responses such as activation of VSMC in order to produce ROS, inflammatory cytokines, chemokines, and adhesion molecules. Changes in angiotensin II production could stimulate the recruitment and activation of myeloid cells initiating local inflammatory response without effect on BP. We aimed to verify if angiotensin II induces an inflammatory response in the aorta and if it correlates with variations in BP. C57Bl/6 mice treated with saline solution (0.9%, control group) or angiotensin II (30ng/kg, Ang II group) were used. BP and HR levels were measured. Immunohistochemistry for IL1-*β*, TGF-*β*, iNOS, CD45, and *α*-actin was performed in the aorta. BP and HR do not change. A biphasic response was observed both for IL1-*β* and TGF-*β* expression and also for the presence of CD45 positive cells, with an acute increase (between 30 and 60 minutes) and a second increase, between 24 and 48 hours. Positive staining for iNOS increased in the earlier period (30 minutes) in perivascular adipose tissue and in a longer period (48 hours) in tunica adventitia. Immunoblotting to *α*-actin showed no alterations, suggesting that the applied dose of angiotensin II does not alter the aortic VSMCs phenotype. The results suggest that angiotensin II, even at doses that do not alter BP, induces the expression of inflammatory markers and migration of inflammatory cells into the aorta of normotensive mice. Thus, angiotensin II may increase the propensity to develop a cardiovascular injury, even in normotensive individuals.

## 1. Introduction

Arterial hypertension characterized by elevated and sustained blood pressure (BP) levels considering BP ≥ 140/90 mmHg is a multifactorial condition and a risk factor for cardiovascular diseases [1–4]. Hypertension is related to alterations in metabolism, hormonal balance and disorders that induce vascular hypertrophy, leading to an increase in peripheral vascular resistance and endothelial dysfunction, which affects macrocirculation [5]. The arteries are formed by three layers: the tunica intima (consisting mainly of endothelial cells), tunica media (consisting mainly of vascular smooth muscle cells), and tunica adventitia (formed by fibroblasts, microvascularization, nerve endings, and inflammatory cells). The interaction between the components present in the blood, the cells that form the vascular wall, and the components of the extracellular matrix (ECM) is important to maintain or change the vascular structure and to determine its functioning and remodeling [6].

It is well known that the redox and inflammatory state and also the renin-angiotensin system are among the many elements responsible for structural changes (arterial remodeling) or functional changes in the vasculature. Previous studies have demonstrated that angiotensin II is one of the main mediators of vascular processes, inducing proliferation and growth of VSMC, collagen deposition, inflammation, and oxidative stress, among others [7, 8]. Inflammatory stimuli are mediated by Ang II through oxidative stress inducing the expression of adhesion molecules and cytokines as well as increasing the recruitment of monocytes [9–12]. In VSMC, Ang II induces the expression of MCP-1 and IL-6. These molecules promote increased infiltration of immune cells by amplifying the inflammatory profile. Thus, Ang II acts as a growth factor that regulates cell proliferation, hypertrophy, and apoptosis, in addition to regulating vascular remodeling [9, 13, 14].

Although many studies have focused on the role of Ang II in the vasculature, these studies involved high doses of angiotensin II, which determined the development of arterial hypertension, and this is recognized as a mechanism directly involved with the activation of inflammatory mechanisms [15, 16]. However, it is not fully understood what proinflammatory effect of angiotensin II would be at concentrations closer to physiological values. In our laboratory, we found that subpressor doses of angiotensin II were able to increase serum IL-6 levels after 60 minutes of a single injection, although not leading to changes in blood pressure [17].

This study aimed to verify the ability of angiotensin II to induce an inflammatory response in the aorta and if there is a relation with variations of arterial pressure, even slight. For this, was evaluated the macrophage infiltration into the aorta and perivascular tissue, in addition to the expression of inflammatory tissue markers in acute or not moments.

## 2. Materials and Methods

The present study used adult male C57Bl/6 mice, weighting between 20g and 25g. The animals were randomly assigned to groups receiving saline solution (0.9%, control group) or angiotensin II (30ng/kg, Ang II group, according to previous study [17]) and evaluated after different times determined in the study. The study was conducted according to the ethical principles established by the Ethics Committee on the Use of Animals (CEUA) of FMUSP, research protocol 184/14.

### 2.1. Direct Hemodynamic Measurement

#### 2.1.1. Catheter Implantation

To verify if the 30ng/kg Ang II dose promotes an inflammatory effect without altering blood pressure (BP) levels, a direct measurement of BP and heart rate (HR) was performed. For this, 16 animals were catheterized, 8 per group, and recorded at the experimental times. The animals were anesthetized with isoflurane for the implantation of a carotid catheter (PE-10, with internal diameter of 0.1mm connected to a PE-50 catheter, internal diameter of 0.5mm). Prior to insertion, the catheters were filled with 0.9% NaCl physiological solution. For implantation of the catheters, a small incision was made in the carotid artery, and the end of the smaller catheter (PE-10) was introduced. The catheters were fixed with cotton thread; the thicker end of the catheter (PE-50) was passed subcutaneously until the neck of the animal, being externalized on the back, near the cervical region. After the surgery, the animals were given analgesic dose (tramadol 40 mg/kg, every 12 hours for 2 days) and antibiotic (Benzathine Penicillin, 50 U/kg, single dose) and were allowed to recover in a warm, O_2_ rich environment, where they were monitored until full recovery. After 48 hours, blood pressure measurements were performed at baseline levels and after administration of Ang II (30ng/kg) or saline solution (NaCl 0.9%) at times 30 min, 1, 2, 6, 12, and 24 h.

### 2.2. Blood Pressure and Heart Rate Assessment

After 48 hours of cannulation, the cannula was connected to an extension of 20 cm (PE-50), allowing free movement of the animal in the box during the experiment period.

The extension was connected to a transducer (Kent Instruments, USA), which was connected to a preamplifier (Hewlet-Packard 8805C, Puerto Rico, USA). The BP signal was recorded on a computer equipped with a data acquisition system (Windak 4KHz, DATAQ Instruments, Akron, OH, USA), allowing beat-to-beat analysis with a sampling frequency of 8000 Hz per channel for studies of systolic blood pressure (SBP), diastolic blood pressure (DBP), mean arterial pressure (MAP), and heart rate (HR). The recording started after the animal has been adapted to the data acquisition system (20 minutes after connecting the animal to the system and when the animal was visually calm). The BP and HR were measured for 20 minutes each time, at the baseline time (prior to the injection, time 0) and after injection of 0.9% saline solution or Ang II (30ng/kg) intraperitoneally, (times: 30 min, 1, 2, 6, 12 and 24 hours). After this, the animals were euthanized with anesthetic overdose of ketamine (180 mg/kg) supplemented with xylazine (20 mg/kg), but there was no collection of tissues of these animals.

### 2.3. Evaluation of Inflammatory Profile

This evaluation was performed in the aorta in order to better understand the vascular modifications induced by Ang II. The animals (7 per group) were treated and euthanized as described previously. After the treatment, the aorta was collected and maintained in buffered formalin 4% for 24–48 h to complete the fixation process. The aorta was processed and paraplast embedded for histological evaluation. Immunohistochemistry for IL1-*β*, TGF-*β*, and iNOS was performed to assess the inflammatory profile, and also CD45 was used as a common marker of macrophages. The evaluation of *α*-actin was performed in order to demonstrate a possible change in the VSMC phenotype. IL1-*β*, TGF-*β*, iNOS, CD45, and *α*-actin expression were measured by immunohistochemistry performed in slices of the aorta incubated with IgG anti-IL1-*β* 1:100 (Santa Cruz Biotechnology, Santa Cruz, CA), anti-TGF-*β* 1:100 (Santa Cruz Biotechnology, Santa Cruz, CA), anti-iNOS 1:200 (BD Transduction Laboratories Biosciences, Franklin Lakes, NJ), anti-CD45 1:100 (Vector Laboratories, Burlingame, CA), and anti-*α*-actin 1:400 (Abcam, Cambridge, MA). Briefly, slices were deparaffinized and rehydrated, and the endogenous peroxidase activity was blocked with hydrogen peroxide (3% in water) for 30 minutes. Following rehydration, the slices were rinsed with phosphate-buffered saline (PBS). The primary antibody was diluted in TBS-TC and applied to the sections for 16-18 hours at 4°C. Subsequently, the samples were washed and incubated with the biotinylated secondary antibody (Zymed Laboratories, South San Francisco, CA) for 60 minutes at room temperature, followed by incubation with streptavidin-peroxidase complex (1: 500) for 60 minutes at room temperature. Finally, a 3,3'-diaminobenzidine solution (DAB, Vector Labs.) was applied. The counterstaining was done with hematoxylin (10 seconds only), and then the slides were mounted. The negative tissue control used in this assay was obtained by omission of primary antibodies during the reaction. At least one cut was maintained as a negative control on each slide assessed. The slices were coded and then studied using an optical microscope (Zeiss Axio Scope II) equipped with a 40x objective and coupled to an image analyzer (Axio Vision 4.8 System).

### 2.4. Immunohistochemistry Analysis

The analyses of IL1-*β*, TGF-*β*, iNOS, and *α*-actin in the tunica media, adventitia, and perivascular adipose tissue (PVAT) were performed by evaluating five photomicrographs of each cut. The ImageJ-Fiji 1.51n Software (Wayne Rasband, National Institute of Health, USA) was used, which has the tool to study immunohistochemistry and allow performing color decomposition. Thus, one can measure the intensity of brown tone obtained by the revelation of DAB. The color intensity values are used for the average calculation of color intensity obtained in each animal. From the mean values of the control group at each time studied (100%), the percentage values of color intensity were calculated in animals treated with angiotensin II. These values were then used for statistical comparison. The analysis for CD45 was performed blindly for treatment and time, by counting the CD45 positive cells in the tunica media, adventitia, and perivascular adipose tissue (PVAT) of five different photomicrographs.

## 3. Statistical Analysis

The statistical analysis was performed by the program GraphPad Prism, version 5.01 (GraphPad Software, Inc., 2007). All values are expressed as means ± SD. These mean values of each animal were compared by Analysis of Variance (ANOVA) of two ways, complemented by the Bonferroni's test. For all statistical analyses, *∗* p≤0.05, *∗∗* p≤0.01, and *∗∗∗* p≤0.001 were considered statistically significant.

## 4. Results

### 4.1. Blood Pressure and Heart Rate Measurement

**Figure 1:**
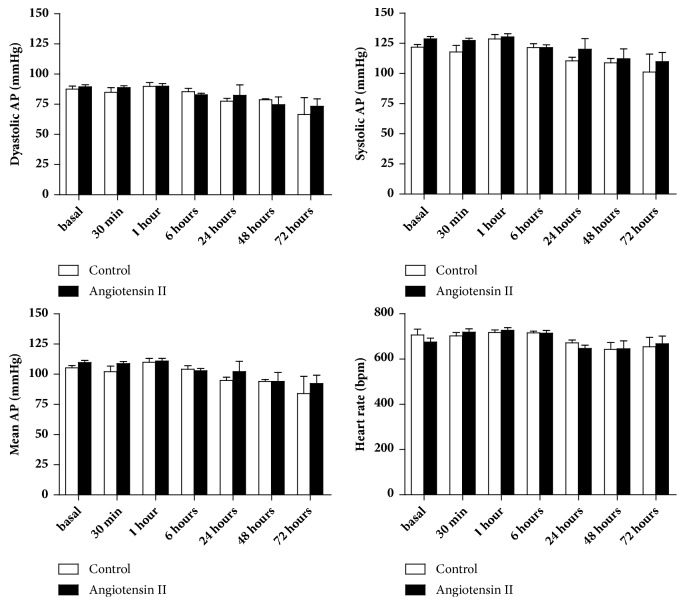
Hemodynamic evaluation showing mean values for dyastolic, systolic, and mean arterial pressure, as well as heart rate. The subpressor dose of angiotensin II did not change any of the evaluated parameters. N=7/group.

Direct hemodynamic measurement of blood pressure and heart rate was performed to verify if the dose of Ang II would change the blood pressure. The dose of Ang II used did not promote any change in blood pressure, diastolic, systolic, or mean, nor of heart rate (Figure 1). In this way, we can consider that changes in inflammatory markers in the aorta are not due to direct blood pressure changes.

### 4.2. Evaluation of Inflammatory Markers

**Figure 2:**
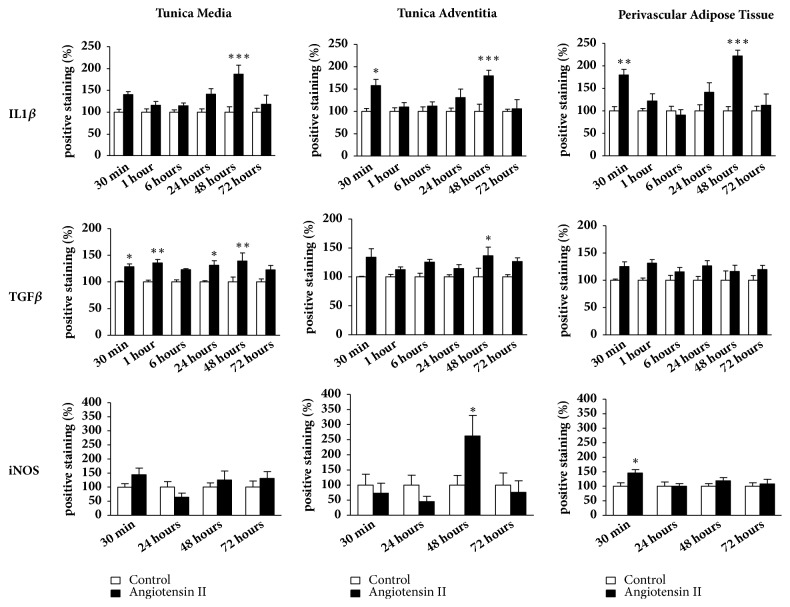
Immunostaining for inflammatory markers in aorta of mice. Positive staining for IL1-*β* increased in earlier period (30 minutes) in tunica adventitia and perivascular adipose tissue (PVAT) and in a long period (48 hours) in tunica media, tunica adventitia, and PVAT after angiotensin II injection. Angiotensin II increased immunostaining for TGF-*β* in acute and late periods in tunica media and later in tunica adventitia. iNOS immunostaining increased in adventitia 48 hours after Ang II injection, while in PVAT increased earlier, after 30 minutes. N=7/group. *∗*≤0.05; *∗∗*≤0.01; *∗∗∗*≤0.001 compared to control.

**Figure 3:**
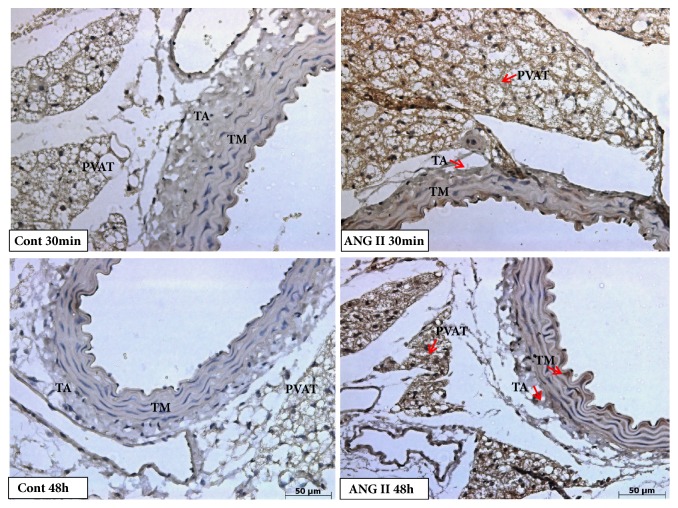
Photomicrography showing immunostaining for IL1-*β* in aorta of mice. Positive staining for IL1-*β* (arrows) increased in earlier period (30 minutes) in tunica adventitia (TA) and perivascular adipose tissue (PVAT) and in a long period (48 hours) in tunica media (TM), tunica adventitia and PVAT after angiotensin II injection (Magnification: 40x).

As previously mentioned, we evaluated IL1-*β*, TGF-*β*, and iNOS as inflammatory markers. The protein expression for IL1-*β* in the tunica media only increased in animals treated with angiotensin II after 48 hours of injection. However, we observed an increase of IL1-*β* expression both in the adventitial tunica and PVAT at two different times, after 30 minutes and 48 hours of Ang II injection (Figures 2 and 3).

**Figure 4:**
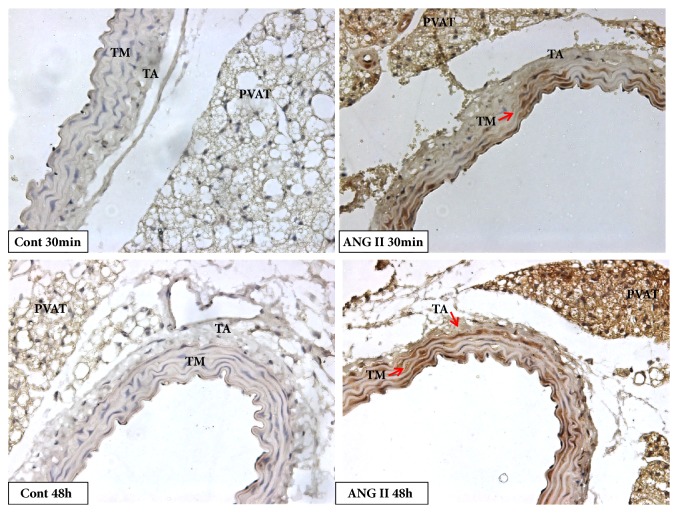
Photomicrography showing immunostaining for TGF-*β* in aorta of mice. Angiotensin II increased immunostaining for TGF-*β* (arrows) in acute and late periods in tunica media and later in tunica adventitia (Magnification: 40x).

The immunostaining for TGF-*β* was positive in the tunica media, with an increase of immunostaining in the angiotensin II group after 30 and 60 minutes of the injection. This increase was maintained until 48 hours after injection (Figures 2 and 4). The adventitial tunica showed a later response, with an increase in the TGF-*β* protein content in angiotensin II only 48 hours after the injection (Figures 2 and 4). On the other hand, there was no increase in TGF-*β* protein expression in PVAT as shown in Figures 2 and 4.

**Figure 5:**
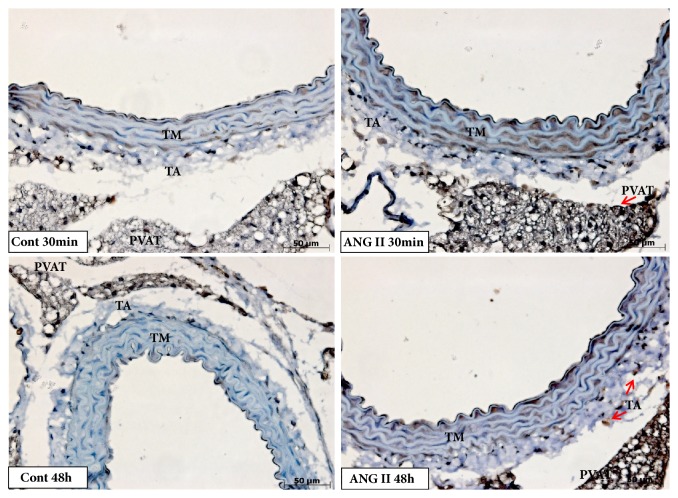
Photomicrography showing immunostaining for iNOS in aorta of mice. iNOS immunostaining (arrows) increased in adventitia 48 hours after Ang II injection, while in PVAT increased earlier, after 30 minutes (Magnification: 40x).

There was no difference of iNOS expression in the aortic tunica media (Figures 2 and 5). However, it was possible to observe an increase of iNOS protein expression in the adventitial tunica after 48 hours of angiotensin II injection (Figures 2 and 5), as well as an increase in PVAT after 30 minutes of injection (Figures 2 and 5).

### 4.3. Evaluation of Macrophage Migration by CD45 Immunostaining

Immunostaining evaluation of the common macrophage marker (CD45) showed that with only a single dose of angiotensin II (30ng/kg) it is possible to observe the recruitment of positive cells in the aorta. This recruitment differs in time after exposure to angiotensin II, as well as to the region of the vessel under study.

**Figure 6:**
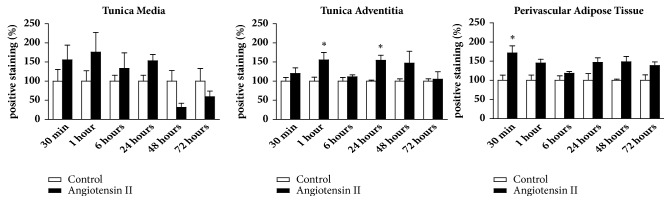
Macrophage counting in aorta of mice. The subpressor dose of angiotensin II (30ng/kg) was able to induce acute macrophage migration, after 30min in perivascular adipose tissue. The injection of angiotensin II was also able to increase macrophage density in adventitia in two different moments after 1 hour and 24 hours. N=7/group. *∗*≤0.05 compared to control.

**Figure 7:**
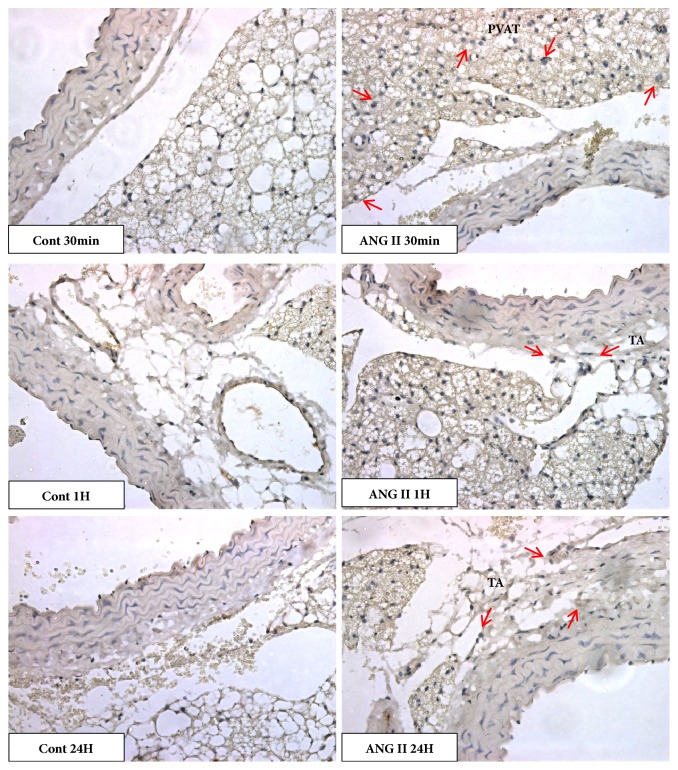
Photomicrography showing immunostaining for CD45 in aorta of mice. The subpressor dose of angiotensin II (30ng/kg) was able to induce acute macrophage migration (arrow), after 30min in perivascular adipose tissue (PVAT). The injection of angiotensin II was also able to increase macrophage density in tunica adventitia (TA) in two different moments after 1 hour and 24 hours (Magnification: 40x).

As can be seen in Figure 6, there was no difference in the population of CD45 positive cells between the groups in the tunica media. On the other hand, there was an increase in the population of CD45 positive cells in the Angiotensin II group in the tunica adventitia (Figures 6 and 7) shortly after 1 hour of the injection; this increase again occurs after 24 hours. In the perivascular adipose tissue (PVAT), the response was even more acute (Figures 6 and 7), with an increase in positive cells after 30 minutes of angiotensin II injection. In addition, in the same way as shown in the adventitial tunica, there is a slight reduction after 6 hours, followed by a further increase of tissue-resident positive cells after 24 hours.

### 4.4. Evaluation of the *α*-Actin in the Tunica Media

As described above, analysis of *α*-actin expression reported the status of smooth muscle cells as to their contractile phenotype. The more intense the labeling, the more likely the contractile cytoskeleton to be well structured, indicating a clearer contractile phenotype. On the other hand, weaker color intensity indicates a probable cytoskeletal dissociation, reducing its contractile capacity, and is more likely to be associated with a synthetic phenotype (which appears in smooth muscle cells activated and involved in a pathological process).

**Figure 8:**
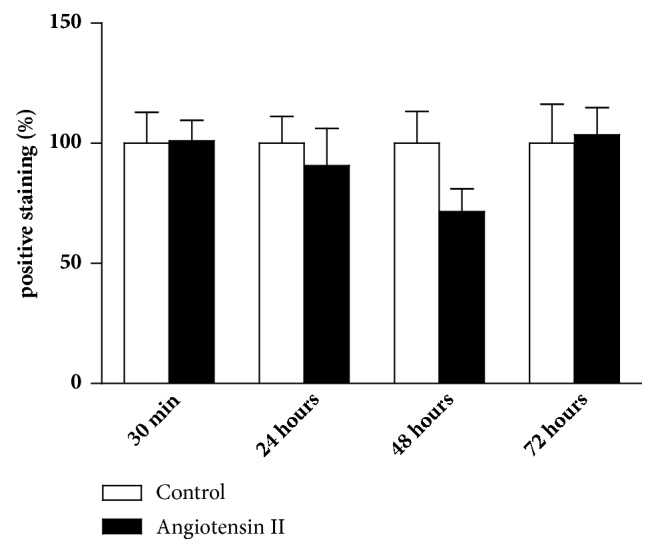
Immunostaining for *α*-actin to study contractile phenotype of vascular smooth muscle cells in tunica media. The analysis showed a slight reduction (not significant) of positive staining in mice injected with angiotensin II, after 48 hours. N=7/group.

**Figure 9:**
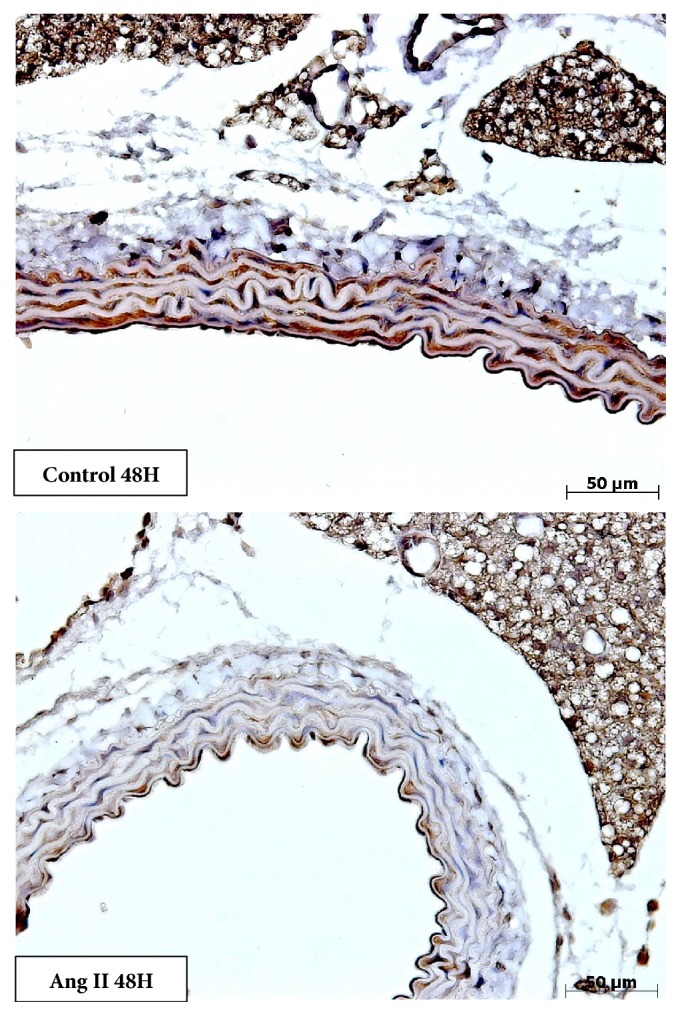
Photomicrography showing immunostaining for *α*-actin to study contractile phenotype of vascular smooth muscle cells in tunica media. There was a slight reduction (not significant) of positive staining in mice injected with angiotensin II, after 48 hours (Magnification: 40x).

The treatment with angiotensin II at a dose of 30ng/kg did not change the *α*-actin expression in the tunica media. Although a reduction of *α*-actin content may be observed after 48 hours (Figures 8 and 9), we did not find statistically significant differences. As shown below, this time of 48 hours corresponds to a time of greater expression of inflammatory markers, which suggests that further investigations are important.

### 4.5. Discussion

In this study, we aimed to demonstrate that angiotensin II, even in small amounts, would activate a vascular inflammatory response. We proposed to start the study by evaluating whether a single dose of angiotensin II (30ng/kg) injected intraperitoneally in mice was effectively subpressor and unable to change blood pressure. The results showed that the injection of angiotensin II, even in a subpressor dose, activates the inflammatory response in the aorta by increasing inflammatory infiltrate and local inflammatory markers.

Previous studies have associated cardiovascular diseases with a chronic state of low grade inflammation. These studies are based on experimental models of hypertension, although clinical studies confirming the involvement of the immune response in cardiovascular disease have increased. The cellular and molecular immunological processes that result in vascular alterations associated with arterial hypertension represent a new paradigm in the research of therapeutic strategies for cardiovascular diseases [18]. As we expected in our study, a single dose of angiotensin II (30ng/kg) did not change blood pressure and heart rate in any of the studied times. Thus, the results obtained in this study are independent of BP and HR changes.

After confirming that the stimulus of Ang II given to the animals did not affect BP, we evaluated if there was a change in the VSMC phenotype at aortic wall. Since those cells switched their phenotype from contractile to synthetic, it participates in the release of several substances such as inflammatory markers. For this, we evaluated the *α*-actin protein content in the tunica media. Previous studies have demonstrated that Ang II was able to reduce *α*-actin levels via the JNK pathway and p38MAPK during hypertension [19]. However, increased levels of *α*-actin in the mesenchymal-endothelial transition in the intraluminal thrombus of abdominal aortic aneurysm were related to a fibrogenic process and to cardiovascular diseases [20].

In our study, we expected a decrease in *α*-actin protein expression in the tunica media of animals treated with angiotensin II. However, there was no significant difference between groups, although there was an apparent decrease after 48 hours of injection. We believe that the stimulation given with the angiotensin dose (30ng/kg) may not have been enough for VSMC initiating a change in phenotype. Although the results demonstrate that there was no change in VSMC phenotype, the evaluation of inflammatory infiltrate and inflammatory markers was important to evaluate the recruitment of immune cells to the aorta.

**Figure 10:**
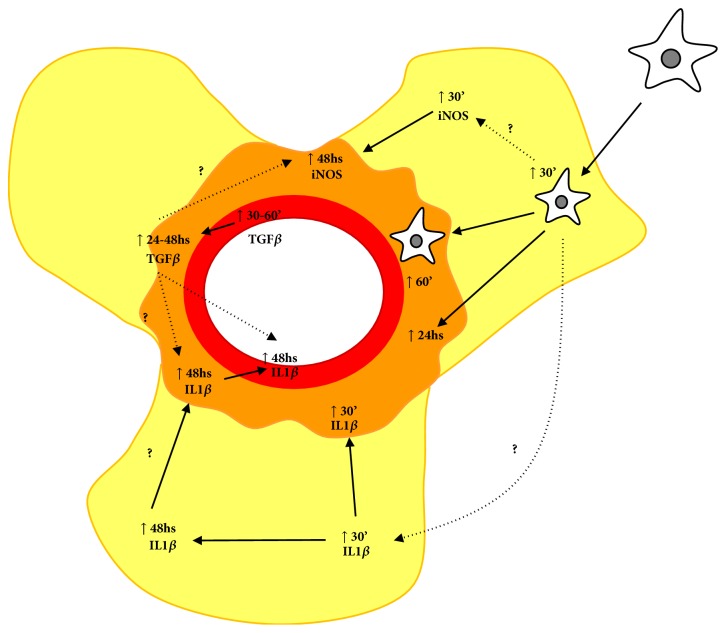
Proinflammatory responses of angiotensin II in aorta of mice. In the presented scheme, Ang II induces a fast macrophage migration to PVAT, possibly increasing iNOS and IL1-*β* expression. A secondary macrophage migration to tunica media may be related to a late increase of iNOS and IL1-*β* expression. On the other hand, TGF-*β* quickly increases in tunica media. The reason why the late expression of TGF-*β* in adventitia and PVAT could be influenced by earlier TGF-*β* expression or by macrophages infiltration needs to be investigated.

Evaluating the presence of CD45-positive myeloid cells in the tunica media and adventitia, as well as in perivascular adipose tissue, we observed that there was no difference in CD45 expression in the tunica media. On the other hand, there was an increase in the presence of CD45 positive cells in PVAT after 30 minutes of injection, and also after 1 and 24 hours in the adventitial tunica. Thus, a single dose of Ang II at the concentration of 30 ng/kg was able to alter the levels of CD45, a common marker of leukocytes evidencing the participation of innate immunity in response to this stimulus. Previous studies have shown that Ang II, the major vasoactive peptide of the renin-angiotensin system, has functions beyond its vasoconstricting activities but also presents proinflammatory characteristics in the vascular wall, inducing the production of inflammatory cytokines, adhesion molecules, and ROS production, resulting in the accumulation of macrophages, differentiation of myofibroblasts [21, 22]. Activated macrophages may play a protective and pathogenic role in several diseases, including vascular diseases. Several studies revealed that macrophages may exhibit mesenchymal cell phenotype under inflammatory conditions [20]. Guzik et al. [22] demonstrated increased levels of CD45 in the aorta of mice treated with Ang II. In addition, Ang II stimulates T cells to produce TNF-*α* and IFN-*γ*. Thus, both innate and adaptive immunity seem to be involved in angiotensin II-induced hypertension and lesions in target organs. [22–24]. In this study, we also have analyzed the positive immunostaining of IL1-*β*, TGF-*β*, and iNOS, important inflammatory markers. Interestingly, the three inflammatory markers showed an acute increase in the aorta (times of 30 to 60 minutes) and, subsequently, a second increase after 48 hours (Figure 10). These results are according to results obtained previously in our laboratory, where a subpressor dose of angiotensin II (30 ng/kg) in normotensive mice increased the expression of TGF-*β* and IL-6 in cardiac arteries and also systemic IL-6 concentration [17]. Recent study demonstrated monocytes and M2 macrophages accumulation in vascular wall after angiotensin II-induced hypertension [25].

Several studies suggested that Ang II is a potent inducer of vascular inflammation, and this has already been observed in Ang II acute infusion models in mice, which demonstrated a large vascular inflammatory response, including increased production of cytokines, chemokines, and ROS in endothelial cells and aortic VSMC prior to aneurysm formation [21, 26]. On the other hand, Nosalski et al. (2017) recently reviewed the mechanisms by which angiotensin II induces inflammatory markers activation, followed by immune cells activation and migration, leading to immune cells accumulation, new cytokine production, tissue remodeling, and the establishing of hypertension [27].

The present study shows for the first time that angiotensin II is capable of inducing migration and accumulation of immune cells in the vascular wall, even without the change in blood pressure. This study also worked to explain the possible steps by which cytokines are produced and the immune cells migrate through the intima to the adventitia and PVAT. Certainly, more studies are needed to understand how Ang II activates this inflammatory mechanism. Possibly, cell culture studies would be instrumental in assessing how VSMC respond to Ang II and how pathways for inflammatory cytokine production are activated. Although we have not observed the reduction of *α*-actin immunostaining, as expected, the decrease observed may represent a stimulus for the VSMC acting as proinflammatory cells. The results suggest that angiotensin II may be considered to be capable of increasing the propensity to develop a cardiovascular injury, even in normotensive individuals.

## 5. Conclusion

The results obtained in the present study suggest that angiotensin II, even without hypertension, acts on the aorta wall in order to stimulate a migration of CD45 positive cells from PVAT, and it stimulates different cytokine production in wall regions. This angiotensin II effects may facilitate a process of tissue injury even in normotensive animals.

## References

[1] Mackay J., Mensah G. A. (2004). *The atlas of heart disease and stroke*.

[2] World Health Organization (WHO) (2002). Integrated Management of Cardiovascular Risk. Report of a WHO Meeting. *Non-communicable Diseases and Mental Health Publication*.

[3] Passos V. M. A. (2006). Hipertensão arterial no Brasil: estimativa de prevalência a partir de estudos de base populacional. *Epidemiologia e Serviços de Saúde*.

[4] World Health Organization (WHO) (2009). *World Health Statistics*.

[5] Taddei S., Virdis A., Ghiadoni L., Sudano I., Salvetti A. (2002). Effects of antihypertensive drugs on endothelial dysfunction: clinical implications. *Drugs*.

[6] Viegas K. A. S., Lacchini S., Krieger J. E. (2008). Injúria Vascula e Reestenose. *Bases Moleculares das Doenças Cardiovasculares – A integração entre a pesquisa e a prática Clínica*.

[7] Schiffrin E. L., Touyz R. M. (2004). From bedside to bench to bedside: role of renin-angiotensin-aldosterone system in remodeling of resistance arteries in hypertension. *American Journal of Physiology-Heart and Circulatory Physiology*.

[8] Paradis P., Schiffrin E. L., DeMello W. C., Frohlich. E. D. (2009). Renin-angiotensin-aldosterone system and pathobiology of hypertension. *Renin Angiotensin System and Cardiovascular Disease*.

[9] Tedgui A., Mallat Z. (2006). Cytokines in atherosclerosis: pathogenic and regulatory pathways. *Physiological Reviews*.

[10] Marchesi C., Paradis P., Schiffrin E. L. (2008). Role of the renin-angiotensin system in vascular inflammation. *Trends in Pharmacological Sciences*.

[11] Schiffrin E. L. (2008). The flame that lights the fire: Oxidative stress, inflammation, and renal damage in angiotensin ii-induced hypertension. *Hypertension*.

[12] George A. J., Thomas W. G., Hannan R. D. (2010). The renin-angiotensin system and cancer: old dog, new tricks. *Nature Reviews Cancer*.

[13] Pacurari M., Kafoury R., Tchounwou P. B., Ndebele K. (2014). The renin-angiotensin-aldosterone system in vascular inflammation and remodeling. *International Journal of Inflammation*.

[14] Brasier A., Brooke J., Tilton R. G., Grundmann R. (2011). Multifaceted role of angiotensin II in vascular inflammation and aortic aneurysmal disease. *Etiology, Pathogenesis and Pathophysiology of Aortic Aneurysms and Aneurysm Rupture*.

[15] Harwani S. C. (2018). Macrophages under pressure: the role of macrophage polarization in hypertension. *Translational Research*.

[16] Jafri S., Ormiston M. L. (2017). Immune regulation of systemic hypertension, pulmonary arterial hypertension, and preeclampsia: Shared disease mechanisms and translational opportunities. *American Journal of Physiology-Regulatory, Integrative and Comparative Physiology*.

[17] Souza De Oliveira T. C., Viegas K. A., Rabechi N. B. (2011). Proinflammatory effect mediated by angiotensin II on cardiac vessels independent of hemodynamic alterations. *Journal of Hypertension*.

[18] Kasal D. A. B., Neves M. F. (2011). Inflamação como mecanismo patogênico na hipertensão Arterial. *Revista do Hospital Universitário Pedro Ernesto*.

[19] Zhou N., Zhang Y., Wang T., He J., He H., He L. (2015). The imperatorin derivative OW1, a new vasoactive compound, inhibits VSMC proliferation and extracellular matrix hyperplasia. *Toxicology and Applied Pharmacology*.

[20] Rao J., Brown B. N., Weinbaum J. S. (2015). Distinct macrophage phenotype and collagen organization within the intraluminal thrombus of abdominal aortic aneurysm. *Journal of Vascular Surgery*.

[21] Ejiri J., Inoue N., Tsukube T. (2003). Oxidative stress in the pathogenesis of thoracic aortic aneurysm: Protective role of statin and angiotensin II type 1 receptor blocker. *Cardiovascular Research*.

[22] Guzik T. J., Hoch N. E., Brown K. A. (2007). Role of the T cell in the genesis of angiotensin II-induced hypertension and vascular dysfunction. *The Journal of Experimental Medicine*.

[23] Shao J., Nangaku M., Miyata T. (2003). Imbalance of T-cell subsets in angiotensin II-infused hypertensive rats with kidney injury. *Hypertension*.

[24] Barhoumi T., Kasal D. A., Li M. W. (2011). T Regulatory lymphocytes prevent angiotensin II-induced hypertension and vascular injury. *Hypertension*.

[25] Moore J. P., Vinh A., Tuck K. L. (2015). M2 macrophage accumulation in the aortic wall during angiotensin ii infusion in mice is associated with fibrosis, elastin loss, and elevated blood pressure. *American Journal of Physiology-Heart and Circulatory Physiology*.

[26] Longo G. M., Xiong W., Greiner T. C., Zhao Y., Fiotti N., Baxter B. T. (2002). Matrix metalloproteinases 2 and 9 work in concert to produce aortic aneurysms. *The Journal of Clinical Investigation*.

[27] Nosalski R., McGinnigle E., Siedlinski M., Guzik T. J. (2017). Novel Immune Mechanisms in Hypertension and Cardiovascular Risk. *Current Cardiovascular Risk Reports*.

